# Dynamics of blood NAD and glutathione in health, disease, aging, and under NAD^+^-booster treatment

**DOI:** 10.1016/j.redox.2026.104343

**Published:** 2026-08-17

**Authors:** Liliya Euro, Kimmo Haimilahti, Sonja Jansson, Saara Forsström, Jana Buzkova, Anu Suomalainen

**Affiliations:** a Research Programs Unit, Stem Cells and Metabolism Research Program (STEMM), Faculty of Medicine, University of Helsinki, Haartmaninkatu 8, Helsinki, 00014, Finland; b NADMED Ltd, Tukholmankatu 8, Biomedicum 2U, Helsinki, 00290, Finland; c Research Programs Unit, Clinical and Molecular Metabolism Research Program (CAMM), Faculty of Medicine, University of Helsinki, Haartmaninkatu 8, Helsinki, 00014, Finland; d HUS Diagnostic Centre, Helsinki, 00260, Finland; e HiLife, University of Helsinki, 00014, Finland

**Keywords:** Redox profiling, Human blood, NAD, Glutathione

## Abstract

Nicotinamide adenine dinucleotides (NAD^+^, NADH, NADP^+^ and NADPH) and glutathione (GSH and GSSG) metabolites are vital regulators of redox state, enzyme functions, and metabolic flux in hundreds of cellular metabolic reactions. NAD^+^/NADH ratio increases during fasting and has been associated with health outcomes in model systems. A low ratio occurs in specific diseases, treated with high-dose B3-vitamin forms, so-called NAD^+^-boosters. Still, neither healthy human values nor NAD^+^ booster-treated or disease-related levels of NAD(P)(H) or glutathione have been established in humans. Here, we report a standardized workflow of enzymatic cycling assays to quantitatively measure NAD^+^, NADH, NADP^+^, NADPH, GSH, and GSSG from a single whole-blood sample. In the blood of a healthy population aged 18-70 years, these metabolites follow a normal distribution and remain unchanged during aging. High-dose nicotinic acid, a B3 vitamin, resulted in 4-6-fold increase of blood NAD^+^ in healthy individuals, suggesting the need for personalized dosing and follow-up in treatment and drug trials. Our data also indicate disease-dependent “redox fingerprints” of NAD and glutathione forms in different degenerative diseases, including cancers. The evidence highlights the potential of redox profiling as an indicator of probable pathology and as a measure of treatment response.

## Highlights

• Blood NAD and glutathione forms were measured in multiple population cohorts.
• Blood redox metabolites in healthy individuals remained stable across age groups.
• Niacin raised blood NAD+ 4-6-fold in a subject-dependent manner.
• Cancer and neurodegenerative diseases showed distinct redox fingerprints.
• Redox profiling may support treatment monitoring and metabolic pathology detection.

## Graphical abstract

**Figure:**
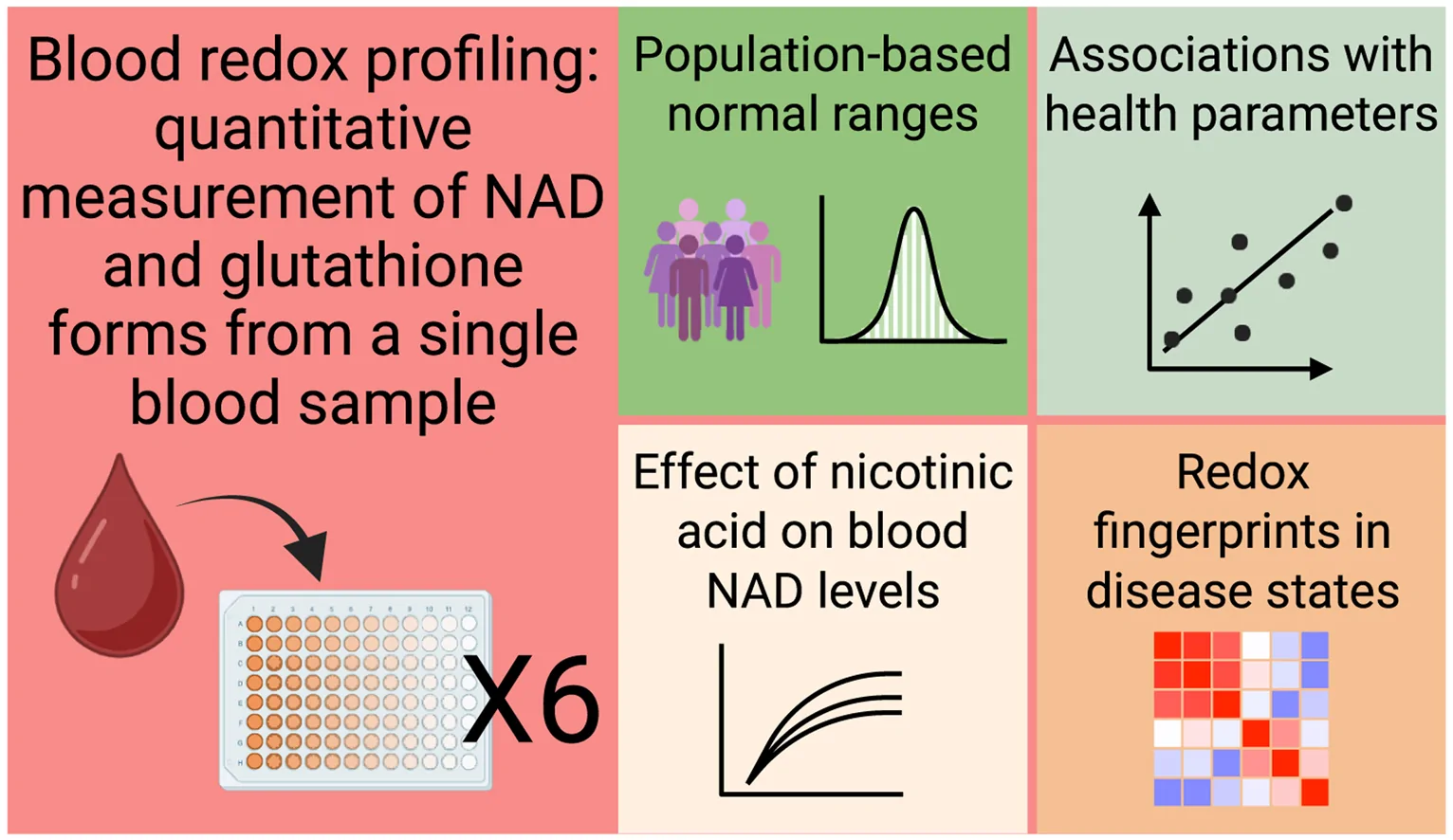

## 1 Introduction

Dysregulation of the physiological redox system is increasingly linked to metabolic imbalance and the development of degenerative diseases, positioning redox biology as a promising translational target. The key redox regulators are the different forms of nicotinamide adenine dinucleotide (NAD) and glutathione. Indeed, altered NAD^+^ and glutathione levels are reported in both genetic metabolic diseases and degenerative, age-related diseases [1,2]. The ability to modulate NAD^+^ and glutathione levels through precursor supplementation has driven growing interest also in aging, longevity, and healthy lifestyle research. However, defining the levels of redox metabolites in healthy populations in clinically accessible body fluids such as blood has been constrained by methodological challenges that limit scalable, high-throughput redox profiling in large population cohorts.

The NAD forms exist as oxidized NAD^+^ and NADP^+^ molecules, which upon accepting two electrons and one proton become reduced to NADH and NADPH, respectively. Both NAD^+^ and NADP^+^ play fundamental roles as electron carriers in cellular metabolism, participating in redox reactions essential for energy production, biosynthesis, and detoxification [[3], [4], [5]]. The major NAD(H)-dependent process in living organisms is a multistep conversion of the energy of nutrients into the high-energy phosphodiester bond of ATP. In addition, NAD^+^ has an essential role as a source of ADP-ribose for three main classes of enzymes, each releasing nicotinamide as a byproduct: 1) mono- and poly-ADP-ribosyltransferases (ARTs, formerly PARPs) utilize ADP-ribose to modify proteins post-translationally and get activated during DNA repair processes [6]; 2) glycohydrolases (e.g., CD38, SARM1, CD157) release free linear ADP-ribose and cyclic cADP-ribose essential for cellular Ca^2+^ signaling [[7], [8], [9]]; 3) sirtuins, the main sensors of cellular nutrient state, are activated by increased levels of NAD^+^ (with the exception of mitochondrial SIRT4, which responds to NAD^+^/NADH ratio) which they use as a co-substrate during deacylation to remove acyl groups from proteins, yielding O-acyl-ADP-ribose [10,11]. Lastly, NAD^+^ serves as a substrate for NAD^+^ kinases, which phosphorylate it to the NADP^+^ form [12]. NADP^+^/NADPH ratio is an essential regulator of the balance between reduced glutathione (GSH) and oxidized glutathione (diglutathione, GSSG) [13]. Glutathione, one of the most abundant cellular metabolites, maintains the cellular environment in a reduced state, protecting proteins and iron-sulfur clusters from oxidative damage, and is essential for detoxification and biosynthetic reactions, including nucleotide synthesis [14]. Both NAD^+^ and glutathione are versatile redox molecules that can function as either a substrate or a cofactor in metabolic reactions. Overall, balanced redox regulation is vital for health across all life forms, and disruption of redox pools is a common consequence of disease.

Levels of different NAD and glutathione forms dynamically respond to exogenous and endogenous changes. Nicotinic acid, a form of niacin (vitamin B3), is a dietary precursor of NAD^+^, and its deficiency causes pellagra [2]. Pathogenic variants in genes encoding NAD^+^-biosynthetic enzymes can also lead to NAD^+^ deficiency [15]. In addition, NAD^+^ deficiency may arise endogenously as a secondary consequence of metabolic disease, for example when disease-related processes increase NAD^+^ consumption or disrupt NAD^+^ homeostasis. Mitochondrial muscle diseases are one such example, with NAD^+^ deficiency detectable in both muscle and blood [16,17]. In these studies, treatment with high-dose niacin restored NAD^+^ levels, providing functional benefits in adult patients: a decrease in liver fat and increases in mitochondrial respiration and biogenesis, muscle strength, and exercise performance [16].

As disrupted NAD and glutathione metabolism become increasingly linked to a range of diseases, they represent compelling targets for medical research. The past decade was marked by expanding development of NAD^+^-boosters, forms of vitamin B3: nicotinamide, nicotinamide riboside, nicotinamide mononucleotide, and their reduced forms, NRH [18] and NMNH [19,20]. These are all derivatives of nicotinic acid, the basic form of vitamin B3. The efficacy of these NAD^+^-boosters in elevating NAD^+^ levels has been extensively studied in model systems and in humans [16,17,19,21,22]. Likewise, N-acetylcysteine (NAC) serves as an alternative source for cysteine and glutathione synthesis [14]. While clinical studies examining the efficacy of NAD^+^-boosters, glutathione, and NAC in various diseases are increasing, uncertainty about normal blood ranges in healthy populations limits the use of four NAD forms and glutathione measurements as clinical trial endpoints. The challenge has been obtaining a comprehensive profile of redox metabolites from a single blood sample, owing to the divergent pH stability requirements of reduced and oxidized species and the high protein content of blood. Although individual NAD metabolites and glutathione can be reliably quantified in deproteinized samples using LC-MS [23], cyclic enzymatic assays, or high-performance liquid chromatography (HPLC) [[24], [25], [26], [27]], scalable high-throughput measurement of all six metabolites from a single sample extract has not been achieved, largely because no extraction method simultaneously preserves the stability of all six metabolites and remains compatible with the detection platform.

Here, we report a quantitative measurement of the major cellular redox regulators (NAD^+^, NADH, NADP^+^, NADPH, GSH, and GSSG) in the blood of several distinct cohorts: a healthy population aged 18-70 years, healthy individuals following vitamin B3 supplementation, and patient groups with age-related diseases. Central to this study is a standardized workflow comprising six enzymatic cycling assays using the same blood extract, which preserves the stability of all six metabolites and is compatible with the assay conditions.

## 2 Results

### 2.1 Analysis of NAD metabolites and glutathione from the whole blood

**Fig. 1:**
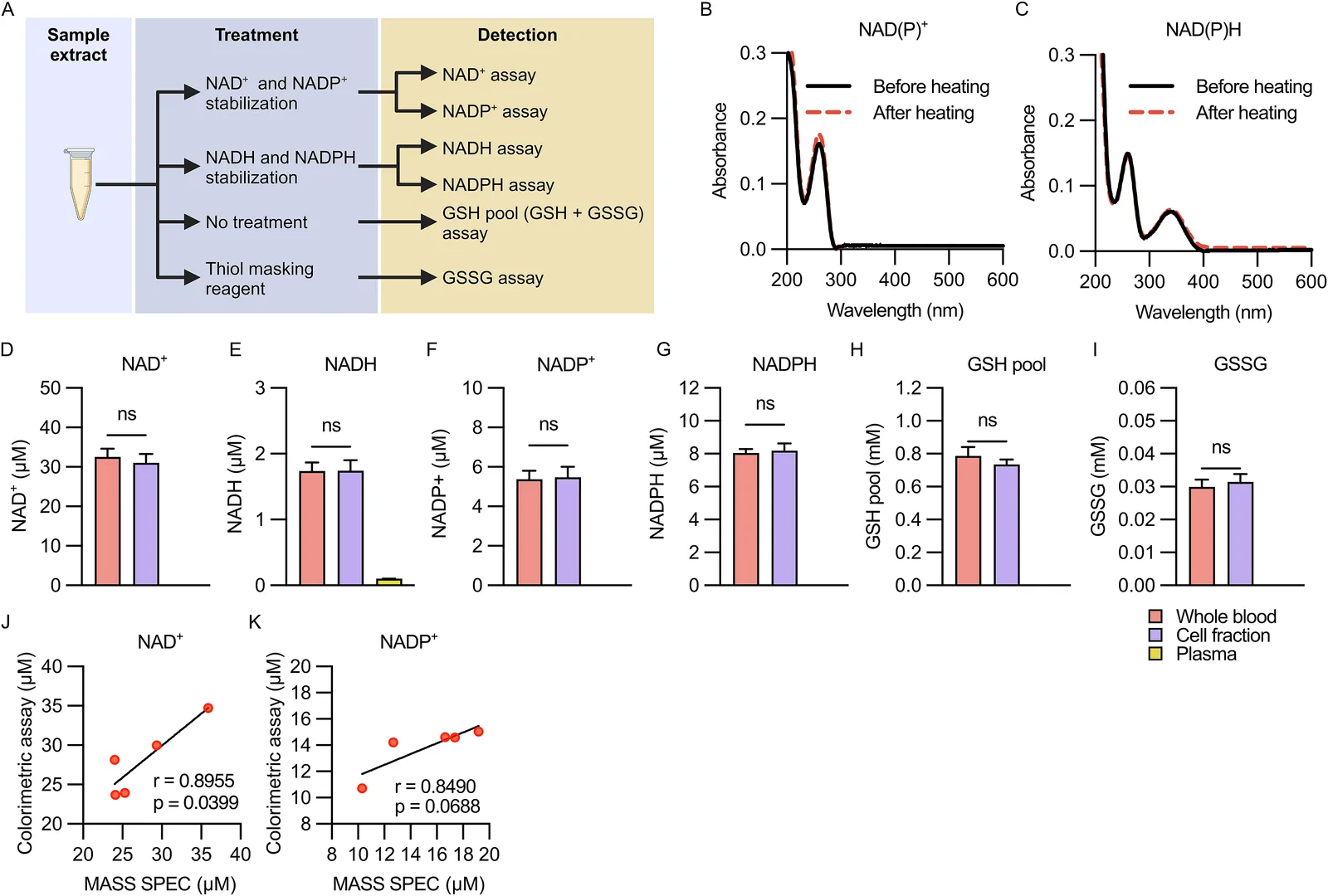
**Validation of a standardized redox profiling workflow for blood**. (**A**) Overview of the redox profiling pipeline. (**B** and **C**) UV-Vis absorbance spectrum of 10 μM NAD(P)^+^ and NAD(P)H in extraction solution before (black line) and after heating (red dotted line). Light absorption spectra of NAD^+^ and NADP^+^ are identical, as are those of NADH and NADPH. (**D** - **I**) NAD metabolites and glutathione in whole blood (n = 3) and its cell and plasma fractions (n = 3) (all samples are prepared from blood of one healthy subject). Data are presented with the mean and standard deviation. (**J** and **K**) Correlation of whole blood NAD^+^ (J) and NADP^+^ (K) between mass spectrometry and enzymatic assays (N = 5, 3 technical replicates per sample). Spearman's correlation is used to calculate correlation coefficients. The difference between whole blood and the blood cell fraction was calculated using the Mann-Whitney nonparametric test.

To quantitatively measure blood NAD^+^, NADH, NADP^+^, NADPH, GSH, and GSSG in large human cohorts, we established a workflow based on commercial kits designed for the analysis of reduced and oxidized NAD forms from a single blood extract. The kits use hot extraction in an alcoholic solution followed by quantification through cyclic enzymatic reactions with colorimetric detection. A key feature of the extraction process is that it keeps proteins soluble while inducing their unfolding and release of all non-covalently bound metabolites, including NAD and glutathione forms, into the extraction solution that preserves the stability of reduced and oxidized redox metabolites. Protein aggregation is then triggered by cooling the homogenate, followed by centrifugation to separate the extract. The extract is then divided into six parts for the individual detection of each target metabolite using a specific enzymatic reaction (Fig. 1A). We verified the stability of NAD^+^, NADH, NADP^+^, and NADPH under the extraction conditions using solutions of pure compounds and monitoring their UV-Vis absorbance spectra (Fig. 1B and C).

To evaluate the extraction efficiency of the six redox metabolites from whole blood and assess potential interference from the biological matrix, we used a spiking approach. Spiking experiments involved adding known amounts of pure NAD compounds or glutathione to fresh and thawed blood and plasma at three points: into the sample before extraction, together with the sample into the extraction solution, and into the hot homogenate before cooling (Table S1), followed by quantification using assays for reduced and oxidized metabolites. In these experiments, 100% recovery of the spiked NAD metabolites was observed only when they were added to a homogenate containing denatured proteins. This indicates the capacity of untreated blood to rapidly hydrolyze certain exogenous NAD forms while actively interconverting others via enzymatic pathways. We observed that the blood plasma exhibits NADH-oxidizing activity, mediating the conversion of added NADH into NAD^+^, while the cellular fraction possesses the capacity to degrade extracellular NAD^+^, NADP^+,^ and NADPH. In contrast, reduced and oxidized glutathione added to blood samples or plasma remained intact. Analysis of different blood fractions revealed that NAD metabolites and glutathione are located within the cells (Fig. 1D–I). Next, we validated NAD^+^ and NADP^+^ measurements in the established workflow using liquid chromatography–mass spectrometry (LC-MS), the recognized reference method for oxidized NAD forms, and found good concordance for NAD^+^ and satisfactory concordance for NADP^+^ (Fig. 1J and K). Because no universally accepted reference method exists for measuring NADH and NADPH in blood, validation of the assays was based on spiking experiments and assessment of reduced metabolite stability under the extraction conditions.

To assess the impact of pre-analytical sample-handling conditions on NAD and glutathione metabolites, we investigated their dynamics in aliquoted blood samples under varying conditions of temperature and time before freezing. Over 24 h at room temperature, NAD^+^ tended to increase by approximately 25% (6.6 μM), NADH by 125% (0.84 μM), and NADPH decreased by 69% (0.74 μM), while NADP^+^, GSH, and GSSG remained stable (Fig. S1, A-F). Extending the incubation to 72 h exacerbated these trends, and even GSH levels fell by 33% and GSSG increased by 81% from the baseline (Fig. S1, G-L). These findings indicate that blood samples undergo dynamic metabolic alterations under *ex vivo* conditions, characterized by distinct changes in the concentrations of individual redox metabolites.

Next, we investigated the effect of a freeze-thaw cycle on blood NAD and glutathione levels. To minimize interference from extracellular enzymatic activity acting on cellular NAD forms released during thawing, we developed a standardized protocol for small-volume frozen samples (150-200 μL): thawing under strictly controlled cooling conditions (+0°C to +2°C) within 12-15 min in an ice-water bath. Freezing did not affect the concentrations of NAD^+^, NADH, GSH, or GSSG. However, it induced conversion of NADPH to NADP^+^, with approximately 50 ± 8% of NADP^+^ derived from NADPH oxidation following a freeze-thaw cycle (Fig. S1, M-R). The mechanism underlying this NADPH-to-NADP^+^ conversion during the freeze-thaw cycle requires further investigation. Using this thawing protocol, we achieved high assay precision, with low intra- and inter-assay coefficients of variation (CV%) for all measured redox metabolites in frozen blood samples (Tables S2 and S3). Assay sensitivities were 0.35 μM for NAD^+^ and NADP^+^, 0.28 μM for NADH and NADPH, 72 μM for the GSH pool, and 10 μM for GSSG (Fig. S1S). These values align with reported ranges for LC-MS and colorimetric methods, confirming this workflow's suitability for quantitative redox profiling in human blood.

### 2.2 Establishment of normal concentrations of NAD and glutathione in whole blood

Prompted by the accuracy of the analytical tools available, we applied our profiling pipeline to determine human population values of the six redox metabolites using frozen whole blood samples from 299 anonymous healthy donors (191 females and 108 males, aged 18-70 years) provided by the Finnish Blood Service BioBank and 38 healthy in-house volunteers (25 females and 13 males, aged 25-70 years). Mean metabolite levels, sample handling, storage details, and criteria for blood donation are summarized in Supplementary Table 4 and in the methods section. We observed that biobank samples (frozen within 11-32 h) and fast-frozen in-house samples (frozen within ∼1 h) showed slight variations in metabolite levels. Specifically, the average NAD^+^, NADH, and NADP^+^ in in-house donor samples were lower compared to biobank donor samples: 24.87 μM vs 28.05 μM, 0.75 μM vs 1.22 μM, and 11.74 μM vs 13.38 μM, respectively (Fig. S2A). In contrast, the average concentration of NADPH in in-house samples was higher than in biobank samples: 2.40 μM vs 1.53 μM, respectively (Fig. S2A). Glutathione levels remained consistent between the two cohorts (Fig. S2A). These results are in line with our observations regarding the dynamics of NAD and glutathione metabolites in blood *ex vivo* (Fig. S1). To account for storage time effects, we combined the Red Cross and in-house samples to obtain control values for frozen blood samples (n = 337). These ranges were derived from mean ± standard deviation (Table 1). The control values are valid for NAD^+^, NADH, GSH, and GSSG for both frozen and fresh blood (within 72 h of storage). However, NADP^+^ and NADPH levels in frozen blood differ from those in fresh blood due to the oxidation of NADPH during the freeze-thaw cycle (Fig. S1). Based on the finding that approximately 50% of NADPH is converted to NADP^+^, we estimated control values for fresh blood NADP^+^ and NADPH using values from frozen samples and validated these calculations by analyzing fresh blood samples from 12 healthy volunteers in our in-house cohort (Table S5). However, fresh blood remains the most suitable sample type for analyzing physiological levels of the phosphorylated forms of NAD metabolites.

**Table 1:** **Physiological levels of four NAD and two glutathione forms measured from frozen blood samples of non-supplemented healthy subjects.** Four NAD forms were analysed from 299 Red Cross blood donors and 38 in-house healthy volunteers. GSH and GSSG were analysed from 298 Red Cross donors and 32 in-house volunteers. For metabolite levels across age and gender groups, and separately for the two study cohorts, see Table S4. Data are mean ± standard deviation.

		Mean	±SD
	Age (years)	18-70	
	N	330-337	
μM	NAD^+^	27.7	6.0
	NADH	1.2	0.4
	NAD pool	28.9	6.2
	NAD^+^/NADH	26.2	9.6

μM	NADP^+^	13.20	2.4
	NADPH	1.6	0.5
	NADP pool	14.8	2.4
	NADP^+^/NADPH	8.5	2.8

mM	GSH	0.76	0.14
	GSSG	0.04	0.01
	GSH pool	0.80	0.15
	GSH/GSSG	18.2	5.7

### 2.3 Redox metabolite concentrations follow normal distribution in healthy population

**Fig. 2:**
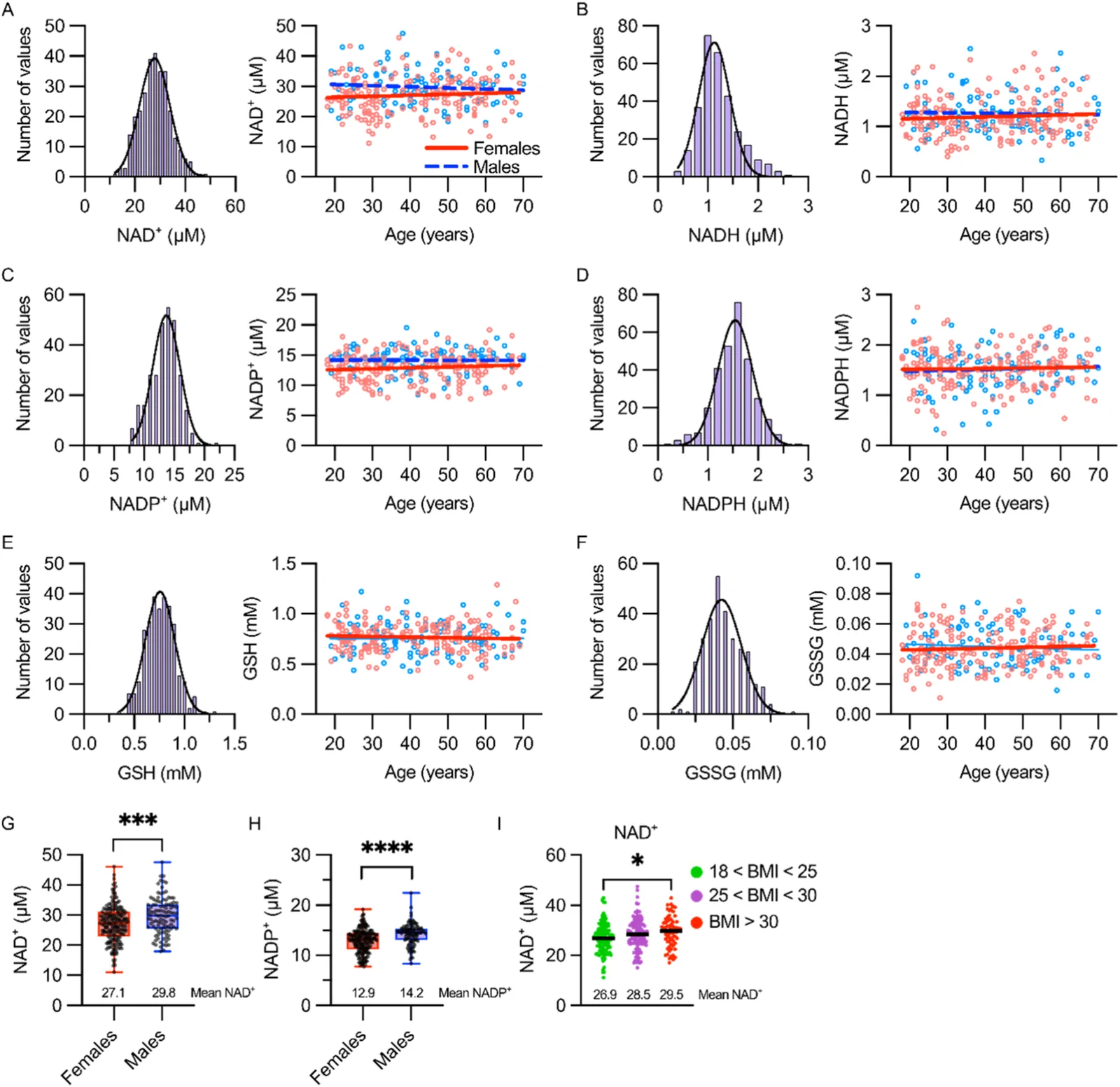
**Blood levels of NAD metabolites and glutathione measured in healthy population remain stable with age and follow normal distribution.** (**A** - **F**) Frequency distribution and age-related levels of NAD^+^ (A), NADH (B), NADP^+^ (C), NADPH (D), GSH (E), and GSSG (F) in the whole blood of 299 healthy donors aged 18-70 years. One sample was lost during extract handling for GSH and GSSG measurements. Red and blue lines represent linear regression fits for females and males, respectively. The black line represents a Gaussian distribution. (**G** and **H**) Blood NAD^+^ and NADP^+^ in females and males. (**I**) Effect of BMI on blood NAD^+^ levels. BMI data were unavailable for two individuals, who were excluded from this analysis. Gender differences were analysed with an unpaired parametric *t*-test. For the effect of BMI, one-way ANOVA with Dunnett's multiple comparison test was applied. *p < 0.05, ***p < 0.001, ****p < 0.0001.

The redox metabolite distributions in the blood samples followed a normal distribution across both genders, with no significant age-related changes in the whole population (Fig. 2A–F; Fig. S2, B-G). When data were grouped by age and gender, mean metabolite values remained largely consistent across the groups with a few exceptions (Fig. S2, H-S). A small but significant difference was observed between females and males aged 18-30 years for NAD^+^, NADP^+^, NADP pool, and NADP^+^/NADPH ratio, with males having higher concentrations (Figs. S2, H, L, N, and O). A similar trend was noted for NADP^+^ and NADP pool in the 51-60 years age group (Fig. S2, L and N). In the 31-40 years age group, males exhibited slightly higher NADH and GSSG levels than females (Fig. S2, I and Q). Interestingly, within this same age group, GSH/GSSG ratio was higher in females than in age-matched males (Fig. S2S). Across the entire cohort, average NAD^+^ and NADP^+^ concentrations were higher in males than females (average difference 2.7 and 1.3 μM, respectively; NAD^+^ males 29,8 ± 6 μM vs females 27,1 ± 6 μM; Fig. 2G and H). In addition, BMI over 30 was associated with an average increase of 2.6 μM in NAD^+^ levels (Fig. 2I). BMI was similar between genders and therefore did not explain the observed differences in NAD^+^ (Fig. S2T). Together, the blood NAD forms and glutathione values obtained from a large population cohort provide a reference framework for interpreting normal and abnormal values in blood samples.

### 2.4 500-750 mg niacin per day saturates steady-state blood NAD^+^ in healthy subjects

**Fig. 3:**
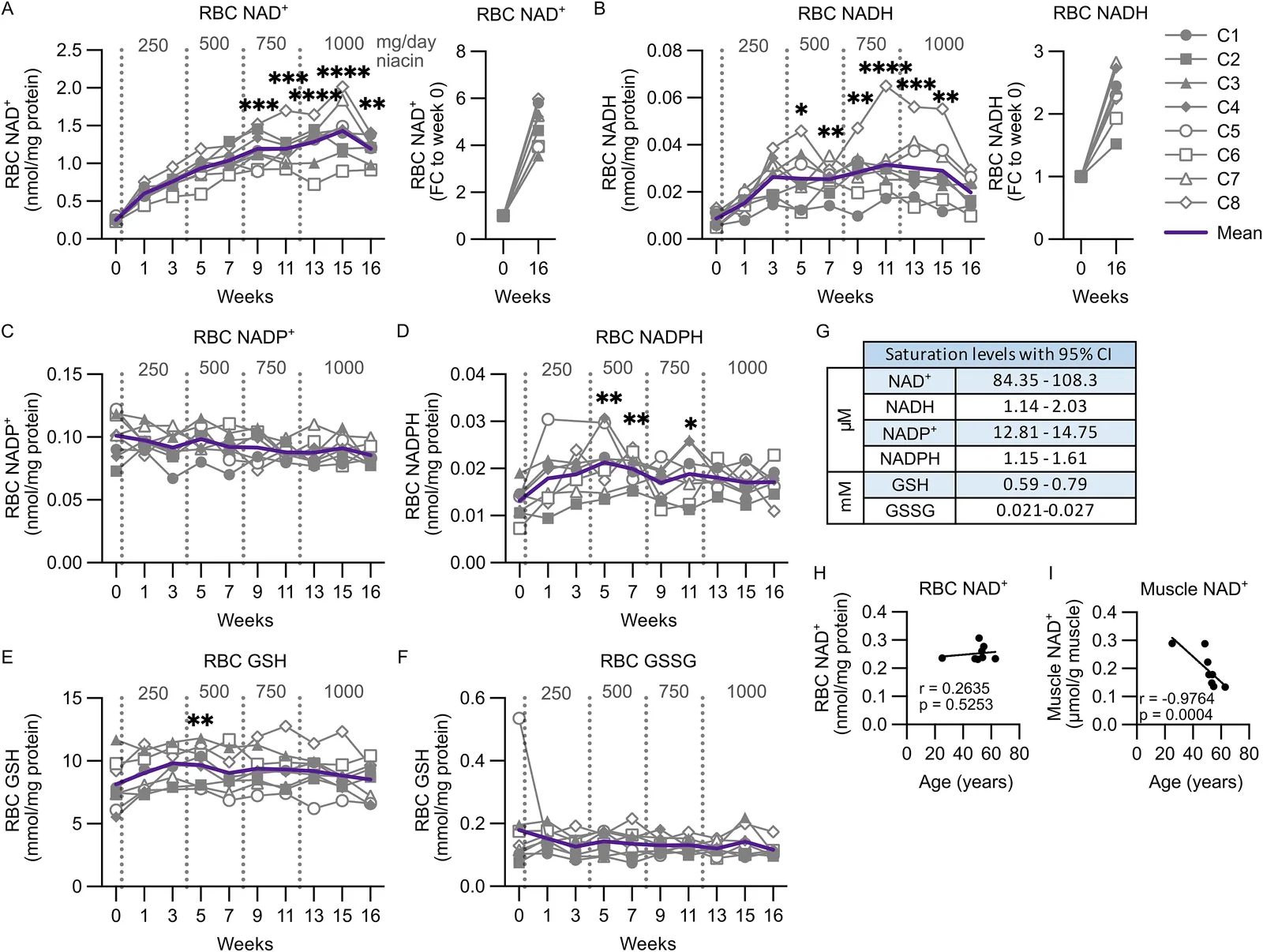
**Red blood cell NAD metabolites and glutathione respond to niacin supplementation in healthy individuals**. (**A** - **F**) Red blood cell NAD^+^ (A), NADH (B), NADP^+^ (C), NADPH (D), GSH (E), and GSSG (F) levels in eight healthy individuals during niacin supplementation. (**G**) Saturation levels of each NAD metabolite and glutathione during niacin supplementation, back-calculated for blood values (see Methods). (**H** and **I**) Spearman rank correlations between age and red blood cell NAD^+^ (H) or muscle NAD^+^ (I). Muscle NAD^+^ was measured by mass spectrometry. Statistical analysis in A-F was performed with Friedman's nonparametric ANOVA with Dunn's multiple comparison test; p-values are against week 0. Data from week 3 was not included in the statistical analysis due to a missing data point. *p < 0.05, **p < 0.01, ***p < 0.001, ****p < 0.0001.

Next, we explored the impact of the NAD^+^ precursor niacin on blood NAD and glutathione metabolites in eight healthy controls. These individuals had served as controls in our previous clinical study, in which nicotinic acid was used as an NAD^+^-booster in patients with mitochondrial myopathy [16]. Their blood samples were collected during the study, and isolated erythrocyte fractions were provisionally biobanked for future studies. At the time, nicotinic acid (form of niacin) was the only NAD^+^-booster with decades of documented human safety data, including at high doses, and was therefore selected for the study. Subjects received increasing doses of niacin (from 250 to 1000 mg daily) for 16 weeks as described previously [16], and blood samples were collected at several time points throughout the trial. Niacin caused marked, dose-dependent increases in erythrocyte NAD^+^ concentrations, reflecting proportional change in whole blood, with subject-dependent kinetics of NAD^+^ accumulation (Fig. 3A). In RBCs, steady-state NAD^+^ reached near-maximal level already at 500 to 750 mg of niacin, and increasing the dose to 1000 mg daily had little or no additional effect (Fig. 3A). Overall, blood NAD^+^ increased 4- to 6-fold by week 16 (Fig. 3A). NADH increased as well but showed substantial inter-individual variation (Fig. 3B). NADP(H) and glutathione remained largely unchanged, although NADPH and GSH showed transient increases after 500-750 mg niacin (Fig. 3C–F). Fig. 3G summarizes saturation levels of all six metabolites back-calculated for whole blood. As in whole blood analysis from the reference population (Fig. 2), erythrocyte NAD^+^ in the eight study subjects did not correlate with age (Fig. 3H). However, their muscle NAD^+^ tended to decrease with age (Fig. 3I). Overall, the results suggest that the niacin dose required to achieve a given steady-state blood NAD^+^ level may vary between individuals.

### 2.5 Specific NAD forms and glutathione correlate with glucose and lipid homeostasis and liver function

**Fig. 4:**
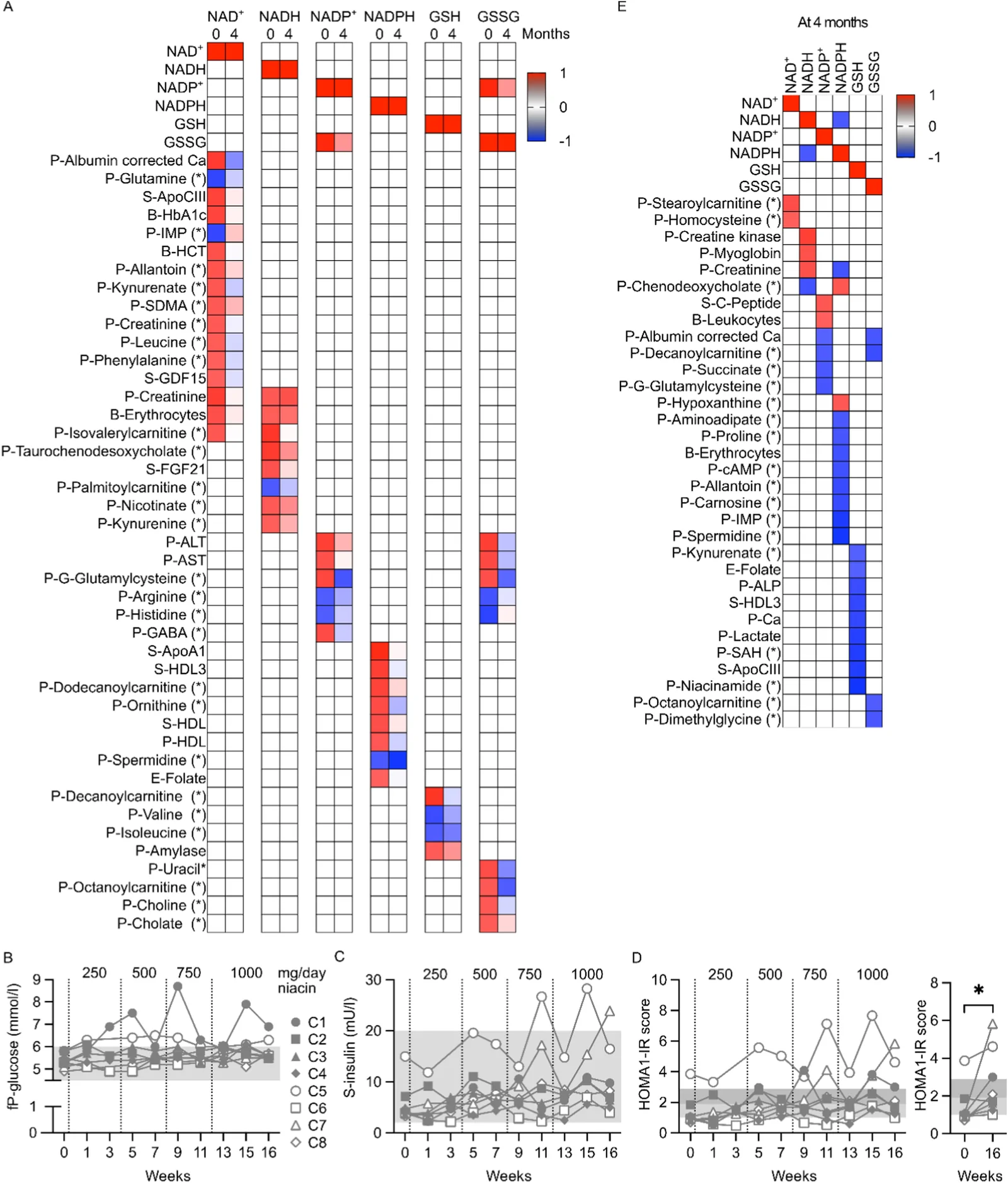
**Glucose and lipid parameters in blood correlate with NAD metabolites during niacin supplementation**. Data are from the eight individuals who received niacin with increasing doses for 4 months. (**A**) Significant correlations of RBC NAD and glutathione metabolites with metabolic blood parameters at baseline and after 4 months of niacin supplementation. (**B** - **D**) Changes in fasting blood glucose (B), insulin (C), and HOMA1-IR (D) in response to increasing niacin doses. (**E**) Correlations of NAD and glutathione metabolites after 4 months of niacin treatment. Spearman's rank correlation was used for association analyses. All correlations shown in panels (A and E) have r > 0.4 and p < 0.05. In panels B-D, the light gray area indicates the normal reference intervals, and in panel D, the dark gray area indicates early insulin resistance (scores 1.9-2.9). Panel D was analysed using the Wilcoxon matched-pairs signed-rank test. *p < 0.05. Sample type: P, plasma; S/not indicated, serum; B, whole blood; RBC, red blood cells. Ca, calcium; ApoA1/CIII, apolipoprotein A1/CIII; HbA1c, glycosylated hemoglobin; IMP, inosine monophosphate; HCT, hematocrit; SDMA, symmetric dimethylarginine; GDF15, growth differentiation factor 15; FGF21, fibroblast growth factor 21; ALT, alanine aminotransferase; AST, aspartate aminotransferase; GABA, gamma-aminobutyric acid; cAMP, cyclic adenosine monophosphate; ALP, alkaline phosphatase; SAH, S-adenosylhomocysteine.

Nicotinic acid as a NAD^+^-booster has been shown to affect a number of health parameters, including blood glucose and cholesterol levels [28]. This prompted us to examine how NAD and glutathione levels correlated with markers of glucose and lipid homeostasis, liver function, B vitamins, and fatty acid metabolism in healthy individuals during the niacin supplementation study. NAD^+^ correlated positively with glycosylated hemoglobin (HbA1c), a measure of long-term blood glucose homeostasis, but the correlation disappeared after 4 months on niacin (Fig. 4A). However, during niacin treatment, fasting glucose, insulin and HOMA1-IR (insulin resistance score based on blood glucose and insulin) increased in some individuals after niacin dose of 500 mg, suggesting trend of increased insulin resistance (Fig. 4B–D). Of metabolic biomarkers, metabokine GDF15 correlated positively with NAD^+^, while FGF21 showed positive correlation with NADH (Fig. 4A). At baseline, HDL cholesterol metrics (total HDL and HDL3) and apolipoprotein A1 (ApoA1) showed positive correlation with NADPH (Fig. 4A), while NADP^+^ correlated positively with liver markers including alanine transaminase (ALT), aspartate transferase (AST) and gamma-glutamyl cysteine (Fig. 4A). Though glutathione forms in RBCs showed less correlation with circulating factors, their GSSG correlated with NADP^+^ and liver markers, including gamma-glutamyl cysteine, which is also a precursor for glutathione (Fig. 4A).

Most of the observed correlations at baseline disappeared after 4 months of niacin treatment (Fig. 4A) with few exceptions. At 4 months, NADH remained positively correlated with creatinine and creatine kinase and myoglobin (Fig. 4, A and E), while NADPH was associated with several metabolites of purine metabolism, such as hypoxanthine, inosine monophosphate (IMP), cyclic adenosine monophosphate (cAMP) and allantoin (Fig. 4E). Of these, IMP and allantoin were also correlated with NAD^+^ at baseline, but not after 4 months on niacin (Fig. 4A). These associations demonstrate a close relationship between the redox metabolites and whole-body metabolism.

### 2.6 Blood NAD and glutathione metabolites are perturbed in various diseases

To explore how chronic age-related diseases modify systemic redox metabolism, we examined blood NAD and glutathione metabolites across 11 different diseases, including various cancer types, type 2 diabetes, and neurodegenerative diseases (Parkinson's and Alzheimer's disease). A total of 164 frozen blood samples were sourced from the Auria BioBank in Turku, Finland, with the majority collected provisionally at the time of diagnosis or treatment initiation (Table S6). However, other additional clinical data, including blood cell count, disease severity, or disease stage at the time of sampling, were unavailable.

**Fig. 5:**
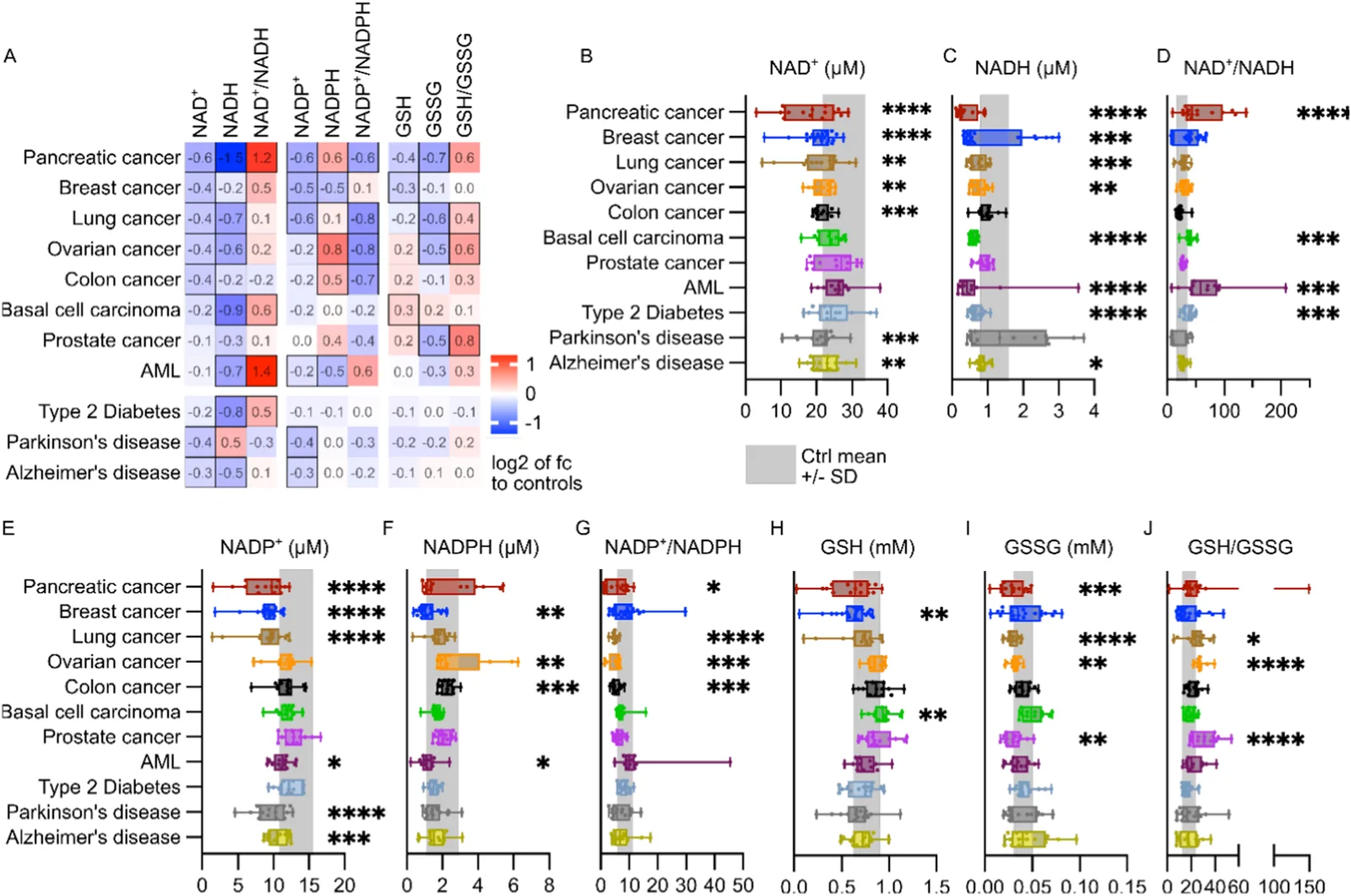
**Diabetes, oncological, and neurological diseases alter blood levels of NAD metabolites, glutathione, and their ratios.** (**A**) Log2 fold changes in whole-blood metabolite levels and ratios relative to the control population (n = 330–337). Statistically significant differences are outlined in black. (**B** - **J**) Absolute whole-blood metabolite levels and ratios across disease groups. The gray area indicates the control range (mean ± SD) defined in Table 1. Statistical analysis was performed using Kruskal–Wallis one-way ANOVA with Dunn's multiple-comparison test versus the control population. *p < 0.05; **p < 0.01; ***p < 0.001; ****p < 0.0001. AML, acute myeloid leukemia.

The changes in individual redox metabolites across different diseases, compared with the reference population, are summarized in the heatmap in Fig. 5A. NAD^+^ and NADH values were low or within the lower end of the healthy range, especially in pancreatic, breast, lung, and ovarian cancer (Fig. 5B and C). As NAD^+^ is the substrate for NADH, the low levels of both metabolites suggest NAD^+^ depletion in these diseases. Likewise, reduced blood NAD^+^ levels were observed in Alzheimer's disease, whereas Parkinson's disease was characterized by decreases in both NAD^+^ and NADH (Fig. 5B and C). NAD^+^/NADH ratio remained unchanged in most diseases but was increased in pancreatic cancer, basal cell carcinoma, acute myeloid leukemia (AML), and type 2 diabetes (Fig. 5D). Notably, AML represents a unique case in this cohort, with the hematopoietic system being the primary tissue affected. This disease is characterized by drastic shifts in blood cell count and an accumulation of immature myeloid cells (blasts), which likely underlie the observed changes in the redox profile. Suggestive subgroups, with either high, low, or unchanged NAD(H), were visible in, for example, breast cancer; however, the limited number of samples and lack of disease-stage data hindered subgroup division. In line with NAD(H) levels, NADP^+^ levels were decreased in several conditions including pancreatic, breast and lung cancers, AML, and Parkinson's and Alzheimer's disease (Fig. 5E). NADPH was increased in ovarian and colon cancer but decreased in breast cancer and AML (Fig. 5F). These changes resulted in decreased NADP^+^/NADPH ratio in pancreatic, lung, ovarian and colon cancer (Fig. 5G). While blood NAD metabolites were altered in all studied diseases, except prostate cancer, glutathione metabolites were altered only in different cancer types (Fig. 5H–J). Among glutathione forms, GSH decreased in breast cancer and increased in basal cell carcinoma (Fig. 5H), whereas GSSG decreased in pancreatic, lung, ovarian, and prostate cancers (Fig. 5I). In the case of lung, ovarian, and prostate cancers, this resulted in an increased GSH/GSSG ratio (Fig. 5J). Overall, blood redox metabolites serve as nonspecific markers of disease.

### 2.7 Correlation heatmaps reveal pathology-related redox fingerprint patterns

**Fig. 6:**
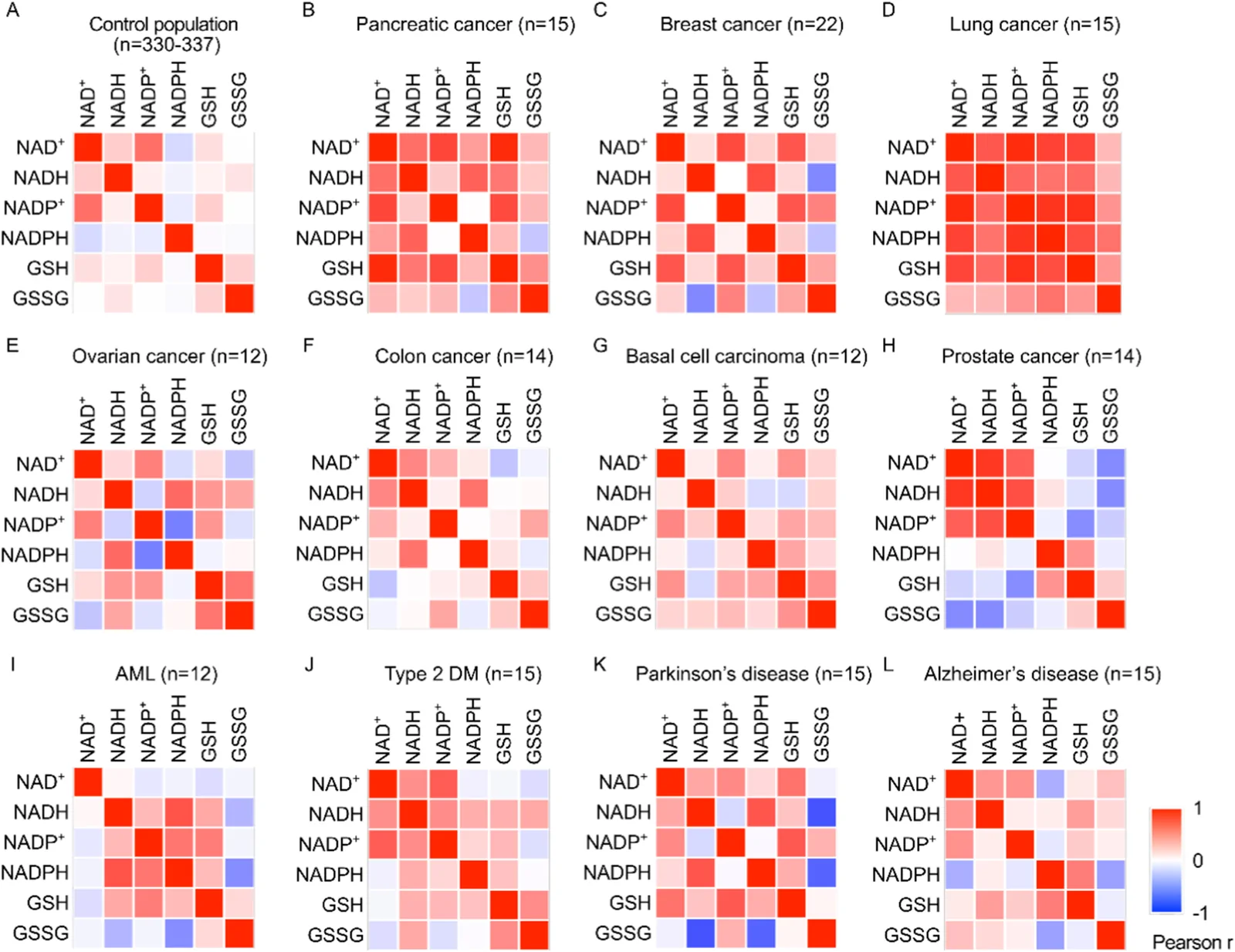
**Disease-specific redox fingerprints differ from the control population and from each other.** (**A** - **L**) Heatmaps of Pearson correlation coefficients showing redox fingerprints of NAD(P)(H) and glutathione profiles in the control population and in the indicated disease groups. AML, acute myeloid leukemia.

Prompted by redox metabolite changes across disease groups, we asked whether the ratios of these metabolites differed from those in healthy controls. The correlation heatmaps – redox fingerprints –visualized the changes in redox state compared to the controls (Fig. 6). In most diseases, NAD^+^ was strongly positively associated with NADH and NADP^+^, consistent with NAD^+^ serving as a substrate for NADH and NADP^+^ synthesis. As an exception, in AML, a hematological malignancy, NAD^+^ did not correlate with any other redox metabolites, potentially because of the altered blood cell composition in this disease (Fig. 6I). While NAD metabolites did not correlate with glutathione in controls (Fig. 6A), in most diseases the correlation was observed. The fingerprints are not disease-specific, but healthy and diseased fingerprints are remarkably different.

## 3 Discussion

Here, we present analysis of NAD^+^, NADH, NADP^+^, NADPH, GSH, and GSSG in a healthy population cohort of blood donors and biobank samples of patients with variable degenerative diseases. We set up and validated a scalable, enzyme-cycling-based standardized workflow that enables accurate quantification of the concentrations of all six redox metabolites from a single fresh or frozen blood sample. We confirmed that the extraction conditions used in the tested method preserve the stability of target redox metabolites, allowing us to perform a comprehensive investigation of these metabolites in blood across different population cohorts. The results show that the blood concentrations of the four NAD and two glutathione forms follow a normal distribution in healthy individuals and remain stable during aging. These findings agree well with previous mass spectrometry-based analysis of NAD^+^ in an aging population [29]. The blood concentrations of each of the six redox metabolites measured in our healthy cohort are consistent with ranges established by diverse methodologies targeting selective redox components [16,[29], [30], [31], [32], [33], [34]]. However, direct cross-study comparisons remain challenging due to highly fragmented literature focused on specific NAD or glutathione form(s) and non-standardized normalization metrics, such as cell volume or cell counts or hemoglobin concentration. A primary strength of our study is that it overcomes these comparative barriers by providing a unified workflow capable of accurately quantifying all six redox metabolites simultaneously from a single whole-blood extract, expressed as standard molar concentrations.

To gain a deeper understanding of tissue-specific NAD^+^ dynamics with age, we utilized available data from our previous clinical trial by performing an updated age-correlation analysis of muscle NAD^+^ in healthy controls [16]. We then paired these results with new measurements from the matching, biobanked frozen blood samples from the same individuals. This cross-sectional analysis indicated no change in blood NAD^+^, contrasting with age-related decline of NAD^+^ in muscle.

Also, some differences between genders exist: males in our large cohort of 299 healthy individuals had, on average, 2.7 μM higher blood NAD^+^ levels than females, consistent with a very similar gender difference in a Chinese cohort with 3.2 μM higher NAD^+^ in males than females [31]. Overall, the similarity of NAD^+^ concentrations between large population cohorts (Finland, Netherlands [29], China [31]), even replicating the gender differences, supports the conclusion that the established healthy ranges are applicable globally.

Our analysis of the dynamics of NAD and glutathione metabolites in withdrawn blood revealed metabolic changes in living cells, showing a transient increase of NAD^+^ and NADH concentrations during storage, which has also been observed previously [32]. These changes are subtle in relation to the baseline concentrations of these metabolites, remain within the physiological ranges determined in two healthy populations, and likely reflect the adaptation of cells to *ex vivo* conditions. Our evidence emphasizes the importance of pre-analytics, specifically the impact of freeze-thaw cycles, which can result in the underestimation of NAD^+^ concentrations. This phenomenon is explained by the loss of cell integrity upon freezing and thawing, which mixes released cellular NAD metabolites with the extracellular compartment containing NAD(P)(H)-dependent enzymatic activities.

The results from hospital biobank samples show that various diseases alter redox metabolite levels and their ratios in blood. Specifically, patients with a range of solid cancer types and neurodegenerative diseases showed decreased blood NAD^+^ concentrations in addition to modified glutathione levels in some cancer types. Our observation of reduced blood NAD^+^ levels and systemic redox imbalance across multiple diseases suggests that diverse pathological processes may drive increased NAD^+^ consumption, potentially through activation of NAD^+^-dependent repair pathways such as PARP enzymes, which are major consumers of NAD^+^ [6]. These results are highly relevant for clinical and research applications, including the monitoring of metabolic interventions and the identification of disease-associated NAD^+^ or glutathione deficiencies to inform patient selection for targeted therapies.

We found that 16 weeks of niacin supplementation in healthy individuals increased blood NAD^+^ levels 4–6-fold in a subject-dependent manner, reaching apparent saturation. A plateau was observed at daily doses of 500–750 mg. Other NAD metabolites showed more modest increases, suggesting differential dynamics of individual metabolite pools. Although niacin supplementation can improve muscle mass and lipid profiles in patients with mitochondrial disease [16] and in the healthy population, high doses of niacin have been associated with hyperglycemia [28] and insulin resistance despite increases in serum adiponectin, which is typically associated with improved insulin sensitivity [35]. Our correlation analysis showed that blood glucose and insulin resistance markers began to increase at niacin doses of 500 mg/day or higher. Notably, glycated hemoglobin (HbA1c), a marker of long-term glycemic control, showed a positive correlation with NAD^+^ levels in healthy individuals already at baseline. These findings suggest that glucose metabolism should be monitored during long-term NAD^+^-boosting interventions. In individuals with elevated NAD^+^ levels, such as those with metabolic syndrome [36], a potential NAD^+^ booster may exacerbate insulin resistance.

While alterations in individual NAD and glutathione metabolites have been reported across various diseases, these findings have historically been limited to localized tissue biopsies or specialized cell types [37]. Consequently, coordinated changes in the systemic redox profile assessed from a single sample remain completely uncharacterized. Our comprehensive whole-blood profiling across various cancer types and age-related neurodegenerative diseases addresses this gap, revealing distinct “redox fingerprints” that distinguish specific diseases from one another and from healthy controls. These observations support further investigation in large biobank cohorts to determine whether such signatures can aid in disease detection or in monitoring treatment responses in clinical trials.

## 4 Summary

In conclusion, our study establishes a conventional approach to quantify the concentrations of four NAD and two glutathione forms in whole blood, demonstrating its applicability to study human redox biology across diverse populations and large sample sets. We have defined baseline reference values for all six redox metabolites in the blood of healthy individuals. Furthermore, we demonstrated that healthy aging is not accompanied by decline in any of these metabolites, whereas pathological conditions induce coordinated shifts in the systemic redox profile. Ultimately, these observed responses to supplementation and disease-associated alterations provide new insights into human redox biology and metabolism.

## 5 Limitations of the study

Reference ranges for NAD and glutathione metabolites were established using blood samples from presumably healthy volunteers. Red Cross samples were collected over three winter months, whereas in-house volunteer samples were collected throughout the year, with repeated sampling in selected individuals. Seasonal variation was not assessed, as the modern Western diet is assumed to provide sufficient vitamin B3 to meet physiological requirements (recommended dietary allowance: 14–16 mg/day). Clinical pathology samples were obtained from the Finnish Auria Biobank retrospective repository and collected over multiple years.

Despite both biobanks utilizing standard operating procedures for blood, sample processing was not uniform across cohorts. Healthy donor blood was initially collected in bags with citrate, then transferred to K2EDTA tubes and stored at ambient temperatures for varying intervals at ambient temperatures followed by incubation at +4°C before freezing. Conversely, patient samples were collected directly into K2EDTA tubes and frozen after generally shorter storage duration. This inconsistency is notable because NAD metabolites fluctuate in viable blood during *ex vivo* storage. Although we quantified these storage-induced alterations in healthy blood, this was not performed for patient cohorts. Consequently, *ex vivo* redox dynamics may distinctively vary depending on the specific disease state.

While we observed stable whole-blood NAD and glutathione levels during healthy aging, these data primarily capture erythrocyte dynamics. Consequently, we cannot rule out localized, age-associated alterations within minor blood cell populations that may be obscured by the overwhelming volume of red blood cells. Normal levels for NAD^+^, NADH, NADP^+^, NADPH, GSH, and GSSG were determined from frozen blood samples of healthy donors. While NAD^+^, NADH, GSH, and GSSG values were considered valid for both frozen and fresh blood samples, concentrations of NADP^+^ and NADPH were found to be affected by the freeze-thaw cycle. To address this, we analysed fresh and frozen aliquots from the same control blood samples, which showed that 50% ± 8% of NADP^+^ in the frozen sample originates from NADPH oxidation during freeze-thaw. This finding allows estimation of NADP^+^ and NADPH levels in fresh blood based on measurements of a frozen sample.

It should be noted that the extraction method used in the kits efficiently releases non-covalently bound metabolites from proteins into solution. Total glutathione in the cells comprises three fractions: 1) bound to proteins through a disulfide bond; 2) free reduced glutathione, and 3) free oxidized glutathione. The obtained extract contains the pool of free glutathione forms. Glutathione covalently bound to proteins remains in the protein pellet post-extraction.

We acknowledge that interpreting our results across different diseases must be done with caution due to limited sample sizes and the lack of information on disease stage in patients with the same disease. Additionally, some diseases, such as certain cancers, are linked to changes in blood cell counts, potentially affecting blood metabolite levels. Our findings warrant further validation in larger data sets.

## 6 Ethical statement and biobank agreements

The Ethics Committee of the hospital district of Uusimaa and Helsinki approved the collection of in-house control blood samples. In-house volunteers who donated blood for test development and validation were from our local community and recruited through an advertisement. All participants gave written consent before blood donation, and samples were anonymized. The human samples were collected by the Red Cross blood donor service (application number #002-2019) and the Aurea biobank (application number AB19-4008) under the biobanks' ethical permits and were shared for this study as part of a research collaboration.

## 7 Materials and methods

### 7.1 Experimental design

The primary objective was to establish method-independent reference levels for six key redox metabolites — NAD^+^, NADH, NADP^+^, NADPH, GSH, and GSSG — in human whole blood using a standardized workflow based on commercially available enzymatic assays and published protocols, thereby supporting reproducibility and broad accessibility. Secondary objectives included 1) exploring dose-dependent effects of niacin supplementation on blood NAD levels to define physiological saturation limits and their associations with biomarkers of glucose and lipid metabolism; and 2) screening disease-associated redox perturbations in patient blood. Previous studies have reported blood levels of selected redox metabolites. Still, a comprehensive redox signature encompassing all six metabolites, quantified from a single blood sample, has not been systematically assessed due to methodological limitations. By confirming previously reported concentrations for metabolites that have been measured before and extending the panel to include additional reduced and phosphorylated forms, this work provides harmonized reference ranges that can be used as a common benchmark across analytical platforms and study designs. We measured each of the six redox metabolites from a single extract obtained through hot extraction of 100 μl of whole blood in an alcoholic solution, which preserves redox-sensitive species and enables selective stabilization and colorimetric detection.

To define robust population reference ranges, whole blood from 300 consecutive, randomly selected healthy blood donors from the Red Cross blood service, aged 18–70 years, was analysed. This unbiased sampling from routine blood donations minimizes selection bias and provides sufficient statistical power for metabolomics studies to characterize normal distributions, age- and gender-related effects, and inter-individual variability.

Additionally, retrospective samples from 8 healthy individuals who received increasing oral niacin doses (250–1000 mg/day for 16 weeks) in a previous clinical pilot [16] were examined. This cohort size, though modest, was deemed sufficient to analyze the effect of increasing niacin doses on NAD^+^ levels in this pilot study and to establish a framework for further studies on individualized NAD^+^-booster dosing.

Using the redox profiling pipeline, we explored disease-specific systemic changes in NAD and glutathione metabolites across 11 disease groups, including several cancers (pancreatic, breast, lung, ovarian, prostate, colon, basal cell carcinoma, and AML) and other diseases (type 2 diabetes, Parkinson's disease, and Alzheimer's disease), analyzing 164 frozen biobank samples from the Auria Biobank. With approximately 15 patients per group, this exploratory study prioritized breadth over depth and was designed to screen for redox perturbations rather than test specific diagnostic hypotheses. This sample size was sufficient to detect large effect sizes and to establish proof-of-concept for distinct disease-associated redox fingerprints relative to healthy controls, supporting the utility of redox profiling as a versatile tool in pathology.

### 7.2 Workflow for quantitative measurement of NAD and glutathione in whole blood

To develop a standardized workflow for quantitative analysis of NAD^+^, NADH, NADP^+^, NADPH, GSSG, and GSH in a single blood sample, we used commercially available blood-specific kits from NADMED and Sigma. For quantitative measurement of NAD^+^ and NADH, we used the Q-NAD NAD^+^ & NADH Blood Kit (REF IVD_001), and for NADP^+^ and NADPH, we used the Q-NAD NADP^+^ & NADPH Blood Kit (REF RUO_004) (both from www.nadmed.com). These kits share the same extraction buffer and extraction procedure but use different detection systems specific either to NAD^+^/NADH or to phosphorylated NAD forms. For measurement of total glutathione (GSH pool) and oxidized glutathione (GSSG), we used the detection system from the Glutathione Assay Kit (Sigma, Cat# CS0260). All kits employ cyclic enzymatic reactions with colorimetric detection in a 96-well plate format.

The NAD measurement kits for blood were stored at −80°C before analysis, whereas the Glutathione Assay Kit was stored at 2–8°C. The Q-NAD kits use hot extraction in an alcoholic solution compatible with the stability of both reduced and oxidized NAD forms, as well as both forms of free glutathione. To extract all six metabolites from a single blood sample, 100 μL of blood was injected into 500 μL of the extraction buffer pre-equilibrated to 75–80°C followed by 1 min incubation of obtained homogenate at this temperature, and then rapidly cooled to precipitate proteins according to kit instructions. After centrifugation at 20 000 g for 10 min at 4°C, the resulting extract was divided into four parts (Fig. 1A).

Each part of the extract was then treated to selectively measure a specific type of redox metabolite, and the treated extracts were used immediately for quantitative analysis in the corresponding enzymatic assay. The first 150 μL aliquot of the blood extract was mixed with 100 μL of NAD(P)^+^ stabilizer containing hydrochloric acid. Acidification of the extract supports stability of oxidized NAD forms and eliminates reduced forms. The compositions of NAD^+^ and NADP^+^ stabilizers are the same in both Q-NAD kits. NAD^+^ and NADP^+^ in the acidified extracts were analysed on separate plates in triplicate, each with its own blanks. In each NAD(P)^+^ assay, 20 μL of acidified extract was mixed with 190 μL of reaction buffer, incubated for 4–5 min at room temperature, stopped by the addition of 10 μL of stop solution, containing sodium dodecyl sulfate, and measured at 573 nm according to the manufacturer's instructions.

The second 150 μL aliquot of the blood extract was mixed with 100 μL of NAD(P)H stabilizer containing sodium hydroxide. Alkalinization of the extract supports stability of reduced NAD forms and eliminates oxidized forms upon exposure to elevated temperature. Therefore, after addition of NAD(P)H stabilizer, the alkalinized extract was heated at 75–80°C for 2 min according to the manufacturer's instructions. The compositions of NADH and NADPH stabilizers are the same in both Q-NAD kits. NADH and NADPH in the alkalinized extracts were analysed on separate plates in triplicate with their own blanks, using the same assay format as for NAD^+^ and NADP^+^ according to the manufacturer's instructions. To account for nonspecific interactions between the sample and the detection system, sample blanks were prepared by omitting the enzyme from the reaction mixture containing the stabilized extracts and all other assay components.

Next, 10 μL of the blood extract t was mixed with 50 μL of deionized water for measurement of the GSH pool. This diluted extract was analysed in triplicate (10 μL per well), each with its own blank, using the Glutathione Assay according to the manufacturer's instructions (Sigma CS0260). The fourth 50 μL aliquot of the blood extract was mixed with 25 μL of a thiol-masking reagent solution containing 2.7 mM M2VP (1-methyl-2-vinylpyridinium triflate, Sigma Cat# 69701) and 32 mM Na_2_CO_3_, followed by incubation for 5 min as previously described [38], and then analysed using the Glutathione Assay (Sigma CS0260) under the same conditions as for the GSH pool, e.g. in triplicate with own blank.

For each metabolite in the original blood sample, the measured concentrations in the treated extracts were corrected for the corresponding dilution factors arising from the extraction and processing steps. Specifically, the dilution factors were 10 for NAD^+^, NADH, NADP^+^, and NADPH; 36 for the GSH pool; and 9 for GSSG. These factors account for the total volume changes during sample preparation, allowing back-calculation of the metabolite concentration in the initial blood volume.

### 7.3 Stability of redox metabolites during extraction process

The stability of NAD^+^, NADH, NADP^+^, and NADPH during extraction was validated using commercially available pure substances (Sigma-Aldrich) (Fig. 1B and C). Concentrations of nucleotides in solutions were monitored by UV-VIS spectroscopy, using extinction coefficients of 18 mM^−1^ cm^−1^ at 260 nm for the oxidized forms and 6.22 mM^−1^ cm^−1^ at 360 nm for the reduced forms [39]. For chemical stability validation, 60 μM stock solutions of each NAD metabolite were prepared in deionized water. Subsequently, 100 μL of each solution was mixed with 500 μL of extraction buffer, heated to 80°C, and then cooled. UV-VIS spectra were recorded in a 10 μM solution before and after heating using a Shimadzu UV-2401PC spectrophotometer.

For validation of matrix effects on recovery of added exogenous metabolites, known amounts of pure NAD and glutathione metabolites were spiked individually at different stages of the extraction of fresh control human blood (Table S1), and recovery of the spiked compounds was monitored in enzymatic assays for both reduced and oxidized forms. When exogenous pure compounds were added to fresh blood just before extraction, recovery rates were below 100%: NAD^+^ at 92.5%, NADH at 10%, NADP^+^ at 80%, and NADPH at 60%. Spiked NADH showed low recovery in reduced form due to oxidation to NAD^+^ as 88% of the spiked metabolite was detected on the NAD^+^ assay plate. Spiking NAD compounds into the extraction solution at the same time as the sample improved recoveries to 98% for NAD^+^, 85% for NADH (with only 10% converting to NAD^+^), 95% for NADP^+^, and 82% for NADPH. When NAD forms were added post-denaturation, 100% recovery was achieved for all NAD compounds. In contrast, reduced and oxidized glutathione (GSH and GSSG) demonstrated high stability with recoveries between 95 and 100% under all tested conditions. Spiking experiments into the plasma fraction before the extraction step revealed that enzymes catalyzing the conversion of NADH to NAD^+^ are localized in plasma, while NAD^+^ degrading activity is associated with cells.

To characterize metabolite compartmentalization, fresh blood samples were fractionated by centrifugation at 1500*g* for 10 min at room temperature. The cell pellets were resuspended in 1× Dulbecco's Phosphate Buffered Saline to a final volume equal to that of the original whole blood sample. Both plasma and cell fractions were analysed using the same workflow as for whole blood, enabling comparison between the components of blood.

### 7.4 Dynamics of redox metabolites in blood *ex vivo*

The behaviour of NAD metabolites and glutathione was assessed in fresh whole blood samples aliquoted into 1.5 mL microtubes and stored at various temperatures for different time intervals. Samples were frozen before the analysis of the metabolite levels.

The effects of freeze-thaw cycles were assessed by analysing blood samples from 12 healthy individuals extracted immediately post-draw and after one freeze-thaw cycle. Based on the results, a conversion factor of 0.5 can be used to estimate fresh blood NADP^+^ from a frozen sample. To estimate fresh blood NADPH, the measured NADPH concentration from the frozen sample can then be added to the value received for NADP^+^.

To minimize the impact of any extracellular NAD(H)-dependent activities on NAD^+^ and NADH levels measured in frozen blood, a standardized thawing protocol was developed: small blood aliquots (150-200 μL) were thawed within 12–15 min in an ice-water bath at +0°C to +2°C, preferably without agitation.

### 7.5 Assay performance, validation of NAD^+^ and NADP^+^ measurements with reference method, compatibility of the extract with LC-MS detection

The pipeline assays demonstrated high precision, with intra-assay coefficients of variation (CV%) ranging from 2.28% to 9.02% and inter-assay CV% from 3.93% to 9.37%. Details on sample numbers, aliquots, and technical replicates are provided in Tables S2 and S3. Detection limits were established as >0.35 μM for NAD^+^ and NADP^+^, >0.28 μM for NADH and NADPH, >72 μM for the GSH pool, and >10 μM for GSSG (Fig. S1S).

Assays for NAD^+^ and NADP^+^ were validated against a targeted quantitative mass spectrometry (MS) method. Parallel frozen blood aliquots from five healthy controls were analysed using kits and an in-house LC-MS NADomics [40]. Correlations showed good concordance for NAD^+^ (r^2^ = 0.90) and satisfactory concordance for NADP^+^ (r^2^ = 0.85) (Fig. 1J and K).

Compatibility of the blood extract used in the pipeline with LC-MS analysis was assessed in MS-Omics facility (Denmark). The set of paired samples, i.e.., frozen blood aliquots and blood extracts from 10 healthy subjects, was sent on dry ice to the MS-Omics facility. Next, blood samples were extracted using their established in-house method for targeted metabolomics [41] (details below in the Targeted metabolomics section) and analysed in parallel with provided alcoholic extracts. Both NAD^+^ and NADP^+^ were detectable from all extracts (fig. S2, U and V).

### 7.6 Targeted metabolomics used for validation

For validation of NAD^+^ and NADP^+^ measurements in blood, a targeted LC-MS NADomics platform developed at the metabolomics facility of the Finnish Institute for Molecular Medicine (FIMM) was employed, as recently described by Presterud et al. [40]. Venous blood from five healthy donors was collected into 2 mL K2EDTA Vacutainers, gently mixed, aliquoted into six microtubes (150 μL each), and frozen at −80°C until analysis. Three aliquots per subject were analysed using the redox profiling pipeline, while the remaining three aliquots were analysed using the targeted LC-MS method. For LC-MS, aliquots were thawed on ice, and 40 μL of whole blood was injected into 450 μL of cold extraction solvent (40:40:20 Acetonitrile:Methanol:Milli-Q water) following the protocol by Presterud et al. [40]. Extracts from each aliquot were analysed in triplicate by both methods. Results were normalized to whole blood volume for comparison.

Metabolomics profiling of extracts obtained by hot alcoholic extraction from Q-NAD kits (www.nadmed.com) was performed as a fee-for-service by the MS-Omics laboratory, Denmark. Ten venous K2EDTA blood samples from healthy donors were collected and aliquoted (two 150 μL tubes per sample) and frozen at −80°C. One aliquot per sample was extracted using the Q-NAD extraction protocol described above, producing frozen extracts. These extracts, along with one frozen whole blood aliquot per donor, were shipped on dry ice to the MS-Omics.

At the MS-Omics facility, frozen blood extracts were analysed directly, while whole blood samples were treated with methanol to precipitate proteins, followed by liquid-liquid extraction with chloroform. The organic phase was used for lipidomics, and the aqueous phase for semi-polar and polar metabolite analysis. Semi-polar metabolite analysis was carried out using a Thermo Scientific Vanquish UPLC coupled to a Thermo Q Exactive HF mass spectrometer in both negative and positive electrospray ionization modes. The UPLC protocol was a slight modification of the protocol described by Doneanu et al. [41]. Compound Discoverer 2.0 software was used for peak extraction. Compound identification was carried out at four confidence levels: Level 1 (matching retention times with authentic standards, accurate mass within 3 ppm, and MS/MS spectra), Level 2a (retention time and mass), Level 2b (mass and MS/MS spectra), and Level 3 (mass only). Polar metabolite analysis followed a similar protocol using the same instrumentation with a UPLC method modified from Hsiao et al. [42]. Identification criteria were applied identically.

### 7.7 Handling of the samples from in-house volunteers

Blood was collected by venipuncture into 2 mL K2-EDTA Vacutainer tubes by an authorized phlebotomist. Aliquots of 200 μL were prepared and placed at −80°C within 30–60 min of collection. Blood from in-house healthy volunteers was used to validate findings observed in the blood donor cohort and to examine redox metabolite properties in blood, as well as the effects of pre-analytical variables on measured values; for cross-check with LC-MS.

### 7.8 Handling of the samples in niacin supplementation study

The study design for niacin supplementation in healthy individuals is described in a previous publication [16]. Blood was collected weekly or biweekly for follow-up measurements into 6 mL K_2_EDTA Vacutainers and separated by Ficoll-Paque density gradient centrifugation according to the manufacturer's instructions (Sigma-Aldrich). The recovered erythrocyte fractions, containing the target metabolites, represented 40–45% of the initial blood volume, yielding concentration factors of 2.2–2.5. Plasma and erythrocyte fractions were collected and stored at −80°C until analysis. Redox profiling of NAD and glutathione metabolites was performed in both fractions using the established pipeline. Erythrocyte results were normalized to protein content (nmol/mg protein), with protein quantified in the cell fraction using the Pierce BCA kit according to the manufacturer's instructions (Thermo Scientific). Initial whole-blood concentrations (pre-density gradient separation) were back-calculated by dividing measured metabolite concentrations (μM) in the erythrocyte fraction by the centrifugation concentration factor. Muscle NAD^+^ and correlation analyses utilized data from our previous study [16].

### 7.9 Sample handling in Red Cross blood service

Whole-blood samples from healthy donors were obtained from the Blood Service Biobank in Finland. Donors were informed about the study and provided written consent before blood donation. A total of 300 samples were collected, but consent for one sample was later withdrawn, resulting in 299 reference samples.

Blood donation in Finland is tightly regulated regarding donors’ health and lifestyle. The list of restrictions is published on the website www.bloodservice.fi. The age of donors ranged from 18 to 70 years. The study cohort comprised consecutive blood donors presenting at the facility on collection days 9.12.2019, 27.01.2020, 4.02.2020, 5.02.2020, and 10.02 -11.02.2021, and who met standard blood bank eligibility criteria (e.g., age, hemoglobin levels, and absence of acute infection risks), with no additional study-specific inclusion or exclusion criteria.

Venous blood was initially drawn into a pouch containing a small amount of citrate. A sample for the study was taken from the pouch into a 4 mL K2-EDTA tube. Samples were transported to the central BioBank facility at ambient temperature over a 3- to 17-h period. Then, a 200 μL aliquot was taken into a small tube, sealed, and kept at +4C° for an additional 8 to 15 h, followed by transfer to a −20C° freezer. In summary, the time interval between blood withdrawal and freezing varied from 11 to 32 h. After keeping the samples for 48 h at −20C°, they were transferred to a −80C° freezer. Samples were delivered on dry ice for analysis. All samples from the Blood Service Biobank were stored as 200 μL aliquots, anonymized, coded, and included data on gender, age, height, and weight.

### 7.10 Sample handling in Turku University hospital Auria BioBank pathology collection

All blood samples were obtained from BioBank's storage after patient informed consent. Venous whole blood samples from patients were collected into 10 mL K2EDTA Vacutainers, centrifuged at 1000g for 10 min at +4°C to collect 1,8 mL of plasma. After removing the target volume of plasma, the samples were carefully mixed again, yielding a 1.22-fold-concentrated blood sample. Next, 400 μL of concentrated blood was pipetted into a small tube, sealed and frozen at −80°C. Reported results are back-calculated to the original blood volume.

The time interval between sample withdrawal and processing ranged from 1.5 to 8 h, with an average of 5 h. During this time, samples were kept in Vacutainers at room temperature. Ten samples differed from the rest, as they were kept for 25-28 h before processing and freezing. Auria BioBank samples were coded and accompanied by information on gender, age, diagnosis, and the time interval between the diagnosis date and sample collection.

### 7.11 Chemicals

All reagents used in the study were from Sigma and of the highest purity available.

### 7.12 Statistical analysis

Data analysis and its graphical representation were done using GraphPad Prism versions 9 and 10. Statistical analyses used are specified in the legend of each figure. Outliers were removed from control data after identification with ROUT Q = 0.5% (number of outliers removed: NAD^+^/NADH ratio n = 2, NADP^+^/NADPH ratio n = 11, and GSH/SSG ratio n = 6; no outliers were identified for individual metabolites).

## CRediT authorship contribution statement

**Liliya Euro:** Conceptualization, Formal analysis, Funding acquisition, Investigation, Methodology, Validation, Writing – original draft, Writing – review & editing. **Kimmo Haimilahti:** Formal analysis, Visualization, Writing – original draft, Writing – review & editing. **Sonja Jansson:** Investigation, Writing – review & editing. **Saara Forsström:** Writing – review & editing. **Jana Buzkova:** Project administration, Writing – review & editing. **Anu Suomalainen:** Conceptualization, Funding acquisition, Resources, Supervision, Writing – original draft, Writing – review & editing.

## Ethical declarations

This study includes human blood and consent was obtained from all donors, or their next of kin or legal representatives, for use in this study and for publication of the article. The privacy rights of participants have been observed. The samples used in this research were not sourced from executed prisoners or prisoners of conscience.

The human samples were collected by Red Cross blood donor service and Aurea biobank according to ethical permits of the biobanks and shared based on research collaboration to this study. The Ethics Committee of the hospital district of Uusimaa and Helsinki approved the collection of inhouse control blood samples.This study was approved by the Ethics Committee of the hospital district of Uusimaa and Helsinki. (Approval No. 110/13/03/01/14).

## Declaration of generative AI and AI-assisted technologies in the manuscript preparation process

During the preparation of this work the author(s) used Perplexity AI and Google AI for language editing to improve sentence formulation. After using this tool/service, the author(s) reviewed and edited the content as needed and take(s) full responsibility for the content of the published article.

## Funding

Funding of this work was supported by Business Finland grant #1498/31/2019, Sigrid Juselius Foundation and the Academy of Finland (for A.S.). Open Access funding for this article was provided by Helsinki University Library.

## Declaration of competing interests

L.E., J.B., and A.S., are co-founders of NADMED Ltd; L.E., J.B., S.J. are employed by NADMED Ltd. and K.H. has received a consultancy fee from NADMED Ltd.

## Data Availability

The de-identified dataset underlying this study are available from the corresponding author on reasonable request.
