# Supplementary material for: Dynamics of blood NAD and glutathione in health, disease, aging, and under NAD+-booster treatment
Source: Redox Biol. 2026 Aug 17;96:104343. doi: 10.1016/j.redox.2026.104343 (PMC13524494; doi:10.1016/j.redox.2026.104343)
Supplement: Supplementary file 1 [file mmc1.docx]

Dynamics of blood NAD and glutathione in health, disease, aging, and under NAD-booster treatment

Supplementary materials

**Supplementary Figure 1. Pre-analytical sample handling alters whole blood NAD(P) and glutathione metabolite levels.**

(A–F) Dynamics of NAD⁺ (A), NADH (B), NADP⁺ (C), NADPH (D), GSH pool (E), and GSSG (F) in aliquoted whole blood during 24 h of storage at room temperature after blood withdrawal (n = 4, 4, 5, 5, 3, and 2 per time point, respectively). (G–L) Dynamics of NAD⁺ (G; n = 18), NADH (H; n = 12), NADP⁺ (I; n = 12), NADPH (J; n = 12), GSH pool (K; n = 6), and GSSG (L; n = 6) in aliquoted whole blood during 3 days of storage at room temperature after blood withdrawal. In all experiments (A–L), samples were frozen before analysis. (M–R) Levels of NAD⁺ (M), NADH (N), NADP⁺ (O), NADPH (P), GSH pool (Q), and GSSG (R) before and after freezing (n = 12). For this experiment, extraction from the first aliquot and freezing of the second aliquot were performed simultaneously within 30 min of blood withdrawal. (S) Practical quantitation limits for each assay. Wilcoxon matched-pairs signed-rank test was used where P values are shown. *p < 0.05; **p < 0.01; ***p < 0.001

**Supplementary Figure 2. Blood NAD and glutathione pools and ratios remain stable with age and follow normal distributions in healthy individuals.**

(A) Whole-blood NAD metabolite and glutathione levels in in-house controls and Red Cross blood donors. The cohorts differed in sampling-to-freezing time: in-house control samples were frozen within 1 h, whereas Red Cross samples were frozen within 10–30 h. (B–G) Frequency distributions and age-related levels of NAD pool (B), NAD⁺/NADH ratio (C), NADP pool (D), NADP⁺/NADPH ratio (E), GSH pool (F), and GSH/GSSG ratio (G) in whole blood from 299 healthy donors aged 18–70 years. Red and blue lines indicate linear regression fits for females and males, respectively. The black line indicates a Gaussian distribution. (H–S) Levels of NAD metabolites, glutathione, their pools, and ratios in whole blood, stratified by sex and age group. (T) BMI in males and females. BMI data were unavailable for two individuals, who were excluded from the analysis. (U and V) Comparison of whole-blood NAD⁺ (U) and NADP⁺ (V) measured by different extraction methods. Metabolite levels in both extracts were measured by the same mass spectrometry facility. In panel A, the Mann–Whitney test was applied. In panels B–T, metabolite levels were measured in 299 healthy blood donors aged 18–70 years; one sample was lost during extraction of GSH and GSSG. Two-way ANOVA with Tukey’s multiple-comparison test was used in panels H–S. An unpaired t-test was used in panel T. Wilcoxon matched-pairs signed-rank test was used in panels U and V . *p < 0.05; **p < 0.01; ***p < 0.001; **** p < 0.0001

| Spike with | | **NAD^+^** | | **NADH** | | **NADP^+^** | | **NADPH** | | **GSH** | **GSSG** |
| --- | --- | --- | --- | --- | --- | --- | --- | --- | --- | --- | --- |
| Average recovery (%) as | | NAD^+^ | NADH | NAD^+^ | NADH | NADP^+^ | NADPH | NADP^+^ | NADPH | GSH | GSSG |
| Sample type | 1. Fresh/thawed blood before extraction | 92.5 | ND | 88 | 10 | 80 | ND | ND | 60 | 95 | 98 |
|  | 2. Fresh/thawed blood during hot extraction | 98 | ND | 10 | 85 | 95 | ND | ND | 82 | 97 | 98 |
|  | 3. Homogenate | 100 | ND | ND | 100 | 100 | ND | ND | 100 | - | - |
|  | 4. Plasma before extraction | 100 | ND | 98 | ND | 100 | ND | ND | 95 | 97 | 100 |

**Supplementary Table 1. Effect of sample matrix and extraction stage on the recovery of spiked NAD and glutathione metabolites.** Pure compounds were spiked into samples at different extraction stages to assess recovery in different matrices. Spiked concentrations were within the physiological range of blood NAD metabolites (1, 4, 10, and 25 mM). Experiments were performed at different times and by two operators. ND, not detected.

|  | Intra-Assay precision | | | | | | | | | | | | | | | | | |
| --- | --- | --- | --- | --- | --- | --- | --- | --- | --- | --- | --- | --- | --- | --- | --- | --- | --- | --- |
|  | **NAD^+^, µM** | | | **NADH, µM** | | | **NADP^+^, µM** | | | **NADPH, µM** | | | **GSH pool, mM** | | | **GSSG, mM** | | |
| Sample ID (n = 10) | 1 | 2 | 3 | 1 | 2 | 3 | 1 | 2 | 3 | 1 | 2 | 3 | 1 | 2 | 3 | 1 | 2 | 3 |
| Mean | 27.41 | 29.41 | 22.00 | 0.55 | 0.71 | 0.64 | 10.03 | 11.59 | 11.01 | 2.39 | 2.57 | 2.62 | 1.03 | 0.86 | 0.76 | 0.03 | 0.022 | 0.036 |
| SD | 0.62 | 1.31 | 0.87 | 0.03 | 0.05 | 0.05 | 0.83 | 0.58 | 0.54 | 0.16 | 0.09 | 0.10 | 0.05 | 0.04 | 0.03 | 0.002 | 0.002 | 0.003 |
| CV (%) | 2.28 | 4.45 | 3.95 | 5.20 | 7.06 | 8.45 | 8.28 | 4.98 | 4.90 | 6.50 | 3.67 | 3.81 | 5.09 | 4.27 | 3.88 | 5.82 | 6.97 | 9.02 |

**Supplementary Table 2. Intra-assay precision for NAD⁺, NADH, NADP⁺, NADPH, GSH pool, and GSSG.** Whole blood from three healthy subjects (sample IDs 1–3) was used. Three aliquots of each sample were extracted, and the resulting stabilized extracts were analyzed in three or four technical replicates for each metabolite.

|  | Inter-Assay precision | | | | | |
| --- | --- | --- | --- | --- | --- | --- |
|  | **NAD^+^, µM** | **NADH, µM** | **NADP^+^, µM** | **NADPH, µM** | **GSH pool, mM** | **GSSG, mM** |
| Mean | 29.36 | 0.76 | 10.91 | 2.35 | 0.782 | 0.031 |
| SD | 1.15 | 0.07 | 0.94 | 0.14 | 0.056 | 0.003 |
| CV (%) | 3.93 | 9.37 | 8.59 | 5.91 | 7.18 | 8.51 |

**Supplementary Table 3. Inter-assay precision for NAD⁺, NADH, NADP⁺, NADPH, GSH pool, and GSSG.** A blood sample from one healthy subject was aliquoted and frozen. Individual aliquots were analyzed in six separate assays for each metabolite, with 10 technical replicates per assay.

|  | | 18-30 years | | | | 31-40 years | | | | 41-50 years | | | | 51-60 years | | | | 61-70 years | | | | RedCross donors | | In-house controls (frozen within 30 – 60 min) | |
| --- | --- | --- | --- | --- | --- | --- | --- | --- | --- | --- | --- | --- | --- | --- | --- | --- | --- | --- | --- | --- | --- | --- | --- | --- | --- |
|  |  | Males | | Females | | Males | | Females | | Males | | Females | | Males | | Females | | Males | | Females | | 18-70 years | | 25-65 years | |
| N | | 22 | | 64 | | 30 | | 33 | | 25 | | 39 | | 20 | | 41 | | 11 | | 14 | | 298-299 | | 32-38 | |
| Metabolite | | Mean | ± SD | Mean | ± SD | Mean | ± SD | Mean | ± SD | Mean | ± SD | Mean | ± SD | Mean | ± SD | Mean | ± SD | Mean | ± SD | Mean | ± SD | Mean | ± SD | Mean | ± SD |
| µM | NAD^+^ | 29.82 | 6.64 | 26.09 | 5.94 | 30.90 | 6.56 | 27.93 | 6.84 | 29.42 | 5.91 | 26.88 | 5.83 | 28.48 | 4.40 | 27.75 | 5.72 | 29.95 | 6.16 | 27.89 | 6.76 | 28.05 | 6.18 | 24.87 | 3.96 |
|  | NADH | 1.22 | 0.41 | 1.21 | 0.36 | 1.35 | 0.39 | 1.05 | 0.31 | 1.20 | 0.30 | 1.18 | 0.37 | 1.27 | 0.49 | 1.26 | 0.41 | 1.20 | 0.45 | 1.25 | 0.42 | 1.22 | 0.39 | 0.751 | 0.288 |
|  | NAD pool | 31.05 | 6.71 | 27.30 | 5.96 | 32.25 | 6.62 | 29.02 | 7.04 | 30.62 | 6.03 | 28.06 | 5.85 | 29.75 | 4.51 | 29.01 | 5.89 | 31.15 | 6.20 | 29.14 | 6.73 | 29.26 | 6.27 | 25.62 | 4.078 |
|  | NAD^+^/NADH | 26.50 | 8.52 | 23.22 | 8.26 | 24.30 | 7.61 | 27.69 | 8.79 | 25.45 | 6.23 | 24.79 | 8.88 | 24.13 | 8.91 | 23.50 | 6.44 | 28.36 | 11.91 | 25.02 | 11.01 | 24.83 | 8.35 | 37.07 | 13.86 |
|  |  |  |  |  |  |  |  |  |  |  |  |  |  |  |  |  |  |  |  |  |  |  |  |  |  |
| µM | NADP^+^ | 14.85 | 2.25 | 12.51 | 2.27 | 14.02 | 2.17 | 13.27 | 2.31 | 13.88 | 2.47 | 12.91 | 2.53 | 14.17 | 1.77 | 12.80 | 2.08 | 14.61 | 1.92 | 14.03 | 2.78 | 13.38 | 2.37 | 11.74 | 1.936 |
|  | NADPH | 1.52 | 0.50 | 1.53 | 0.45 | 1.49 | 0.42 | 1.51 | 0.26 | 1.47 | 0.40 | 1.50 | 0.35 | 1.61 | 0.43 | 1.59 | 0.33 | 1.57 | 0.30 | 1.53 | 0.47 | 1.53 | 0.39 | 2.408 | 0.530 |
|  | NADP pool | 16.36 | 2.10 | 14.04 | 2.37 | 15.51 | 2.27 | 14.78 | 2.28 | 15.34 | 2.53 | 14.42 | 2.58 | 15.78 | 1.78 | 14.40 | 2.19 | 16.18 | 1.97 | 15.56 | 2.71 | 14.91 | 2.41 | 14.15 | 2.007 |
|  | NADP^+^/NADPH | 10.01 | 3.28 | 8.46 | 2.65 | 9.70 | 2.77 | 8.78 | 2.03 | 9.03 | 1.75 | 8.67 | 2.53 | 9.54 | 3.32 | 8.31 | 2.00 | 9.56 | 2.11 | 9.43 | 4.17 | 8.87 | 2.47 | 4.786 | 0.940 |
|  |  |  |  |  |  |  |  |  |  |  |  |  |  |  |  |  |  |  |  |  |  |  |  |  |  |
| mM | GSH | 0.77 | 0.19 | 0.77 | 0.13 | 0.75 | 0.17 | 0.77 | 0.12 | 0.75 | 0.13 | 0.77 | 0.12 | 0.73 | 0.14 | 0.73 | 0.15 | 0.78 | 0.17 | 0.80 | 0.21 | 0.76 | 0.15 | 0.748 | 0.135 |
|  | GSSG | 0.04 | 0.01 | 0.04 | 0.01 | 0.05 | 0.01 | 0.04 | 0.01 | 0.04 | 0.01 | 0.05 | 0.01 | 0.04 | 0.01 | 0.04 | 0.01 | 0.05 | 0.01 | 0.05 | 0.01 | 0.04 | 0.01 | 0.04 | 0.01 |
|  | GSH pool | 0.82 | 0.19 | 0.82 | 0.13 | 0.79 | 0.17 | 0.81 | 0.12 | 0.79 | 0.14 | 0.82 | 0.13 | 0.77 | 0.15 | 0.78 | 0.15 | 0.83 | 0.18 | 0.85 | 0.21 | 0.81 | 0.15 | 0.79 | 0.14 |
|  | GSH/GSSG | 17.18 | 4.90 | 18.88 | 6.42 | 17.46 | 5.87 | 21.16 | 6.73 | 17.72 | 5.43 | 16.99 | 4.72 | 18.51 | 4.12 | 17.73 | 6.10 | 17.63 | 4.51 | 17.78 | 5.16 | 18.26 | 5.71 | 17.35 | 5.251 |

**Supplementary Table 4. Mean values and standard deviations of all measured metabolites in blood samples from 299 Red Cross blood donors and 38 in-house controls.** For GSH and GSSG, 298 Red Cross donor samples and 32 in-house control samples were available for analysis.

|  |  | Physiological levels in fresh blood estimated from measurement of frozen samples | In-house controls (fresh) |
| --- | --- | --- | --- |
|  | N | 337 | 12 |
| µM | NAD^+^ | 21.7 - 33.7 | 24.1 - 30.44 |
|  | NADH | 0.8 - 1.6 | 0.59 - 1.06 |
|  | NAD pool | 22.7 - 35.1 | 23.53 - 32.67 |
|  | NAD^+^/NADH | 16.6 - 35.8 | 25.03 - 45.03 |
|  |  |  |  |
| µM | NADP^+^ | 4.9 - 7.9 | **4.91 -6.91** |
|  | NADPH | 6.6 - 10 | **7.08 - 8.38** |
|  | NADP pool | 12.1 - 17.3 | **12.24 - 15.04** |
|  | NADPH/NADP^+^ | 1.19 - 1.42 | **1.12-1.54** |
|  |  |  |  |
| mM | GSH | 0.62 - 0.91 | 0.64 - 0.94 |
|  | GSSG | 0.03 - 0.05 | 0.03 - 0.05 |
|  | GSH pool | 0.65 - 0.95 | 0.67 - 0.99 |
|  | GSH/GSSG | 12.5 - 23.9 | 16.4 - 28.4 |

**Supplementary Table 5. Reference ranges for redox metabolites in fresh blood.** The table shows that NAD⁺, NADH, GSH, and GSSG concentrations measured in frozen blood are comparable to those in fresh blood, whereas NADP⁺ and NADPH are affected by partial interconversion during freezing, with NADPH contributing to NADP⁺ formation. Based on the observed conversion rate, fresh-blood NADP⁺ and NADPH concentrations were estimated from measurements of frozen samples. The rightmost panel shows values from 12 in-house controls whose blood samples were analyzed fresh to validate these estimates.

| **Diagnosis** | **ICD-10** | **n** | **Days from diagnosis to sampling** | | | | | | | |
| --- | --- | --- | --- | --- | --- | --- | --- | --- | --- | --- |
|  |  |  | **-450...-250** | **-249...-50** | **-49…-1** | **+1...+35** | **+36...+100** | **+101...+250** | **+251...+450** | **+451...+4050** |
| Malignant neoplasm of pancreas | C25 | 15 |  | 2 | 2 | 8 |  | 3 |  |  |
| Malignant neoplasm of breast | C50 | 22 |  | 7 | 15 |  |  |  |  |  |
| Acute myelogenous leukemia (AML) | C92.0 | 12 |  |  |  |  |  | 2 | 1 | 9 |
| Diabetes type II | E11 | 15 |  | 5 | 10 |  |  |  |  |  |
| Parkinson disease | G20 | 15 |  | 5 |  | 1 | 4 | 4 | 1 |  |
| Alzheimer disease | G30 | 15 |  | 4 | 4 | 1 | 4 | 2 |  |  |
| Prostate adenocarcinoma | C61 | 14 |  | 5 | 9 |  |  |  |  |  |
| Skin malignancy | C44 | 15 |  | 8 | 7 |  |  |  |  |  |
| Ovarian epithelial cancer | C56 | 12 | 2 | 2 | 5 | 1 | 1 | 1 |  |  |
| Malignant neoplasm of colon | C18 | 14 |  | 6 | 8 |  |  |  |  |  |
| Malignant neoplasm: upper lobe, bronchus or lung tumor | C34.1 | 15 |  | 4 | 11 |  |  |  |  |  |
|  | Total | 164 | 121 | | | 43 | | | | |

**Supplementary Table 6. Timing of blood sample collection relative to disease diagnosis.** Disease groups represented only by pre-diagnosis samples are highlighted in light gray.
